# Cattle-Related Trauma: A 6-Year Retrospective Study of Patients Admitted to a Trauma Center in China

**DOI:** 10.1155/emmi/7266303

**Published:** 2025-07-12

**Authors:** Shilan Luo, Yuanyuan Zhang, Qi Li, Li Yang, Baosheng Yang, He Jin

**Affiliations:** ^1^Department of Burns and Plastic Surgery, 926th Hospital of Joint Logistics Support Force of PLA, Affiliated Hospital of Kunming University of Science and Technology, Kaiyuan, Yunnan, China; ^2^Department of Disease Control and Prevention, 926th Hospital of Joint Logistics Support Force of PLA, Affiliated Hospital of Kunming University of Science and Technology, Kaiyuan, Yunnan, China; ^3^Department of Foreign Languages, School of Basic Medical Sciences, Army Medical University, Chongqing, China; ^4^Information Department, 926th Hospital of Joint Logistics Support Force of PLA, Affiliated Hospital of Kunming University of Science and Technology, Kaiyuan, Yunnan, China; ^5^Department of Cardiothoracic Surgery, 926th Hospital of Joint Logistics Support Force of PLA, Affiliated Hospital of Kunming University of Science and Technology, Kaiyuan, Yunnan, China

**Keywords:** animal-related injury, cattle, injury characteristics, outcomes, trauma

## Abstract

**Background:** Cattle-related trauma is common in rural areas and is a significant cause of morbidity and mortality, often requiring hospital admission and surgical treatment. However, no literature is currently available on cattle-related trauma in China. We reviewed all patients with cattle-related trauma admitted to a trauma center in China over 6 years and aimed to explore the injury characteristics and outcomes of this trauma.

**Methods:** A retrospective cohort study was conducted, and patients with cattle-related trauma admitted from October 1, 2016, to September 31, 2022, were screened in the Hospital Information System. Demographic data, injury data, clinical treatment information, and outcomes were collected from the medical records and analyzed.

**Results:** A total of 243 patients, with a median age of 48 years (interquartile range [IQR] 31–57), were identified. Of these, 67.5% (*n* = 164) were male and 88.1% (*n* = 214) were farmers. Traveling in a bullock cart as a passenger (31.7%, *n* = 77) was the most frequent injury situation, and bullock cart accident (57.6%, *n* = 140) was the most common injury mechanism. Blunt trauma occurred in most patients (86.4%, *n* = 210). The most commonly injured body region was the lower extremity, pelvis and buttocks (38.3%, *n* = 93). Among the patients, 111 (45.7%) had at least one abbreviated injury scale (AIS) of ≥ 3. Overall, the median injury severity score (ISS) was 8 (IQR 4–13), and 39 patients (16.0%) had an ISS ≥ 16. In total, 209 patients (86.0%) underwent surgical treatment, and 69 (28.4%) were admitted to the intensive care unit (ICU). The median length of hospital stay (LOS) was 10 days (IQR 6–18), and the mortality rate was 1.2% (*n* = 3).

**Conclusions:** This study demonstrated the characteristics of cattle-related trauma in a trauma center in China. Our results may contribute to the development of data-driven safety measures to reduce the risk of cattle-related trauma.

## 1. Introduction

Cattle are common livestock in rural communities; however, the behavior of these large, strong animals is unpredictable [1–3]. Cattle are reportedly responsible for a significant proportion of animal-caused injuries among people who work in agriculture and animal husbandry [4, 5]. Cattle handling is a dangerous activity, and farmers are at risk of developing such injuries [5, 6]. A study of 6438 animal-related on-farm injuries among youth in the United States in one year showed that 31% of these injuries were caused by cattle [7]. Another survey conducted in the United Kingdom that received 2439 questionnaires showed that 24% of farmers reported sustaining cattle-related injuries during handling cattle [8]. Other studies show that cattle account for more occupational fatalities than any other animal [3, 9], and fatalities in cattle-related accidents have been considered to be associated with aggressive behavior of the animal [10]. Cattle-related injuries occur most frequently in farmers; however, nonfarming members of the public are also at risk of cattle attacks when using public footpaths through farms or walking across fields of cattle [2, 11, 12]. Therefore, cattle-related injuries are considered a public health risk in some reports [2, 11].

Cattle-related trauma is considered a significant cause of extreme morbidity and mortality, often requiring hospitalization and surgery [11]. One study that retrospectively reviewed all cattle-related injuries over 5 years presenting to a single trauma center found that 70% of emergency referrals required hospital admission, and 50% underwent operative treatment [11]. Another study demonstrated that 54.9% of hospitalizations for animal-related injuries were caused by cattle [13].

To date, no studies have evaluated cattle-related trauma in China. This retrospective study aimed to determine the injury characteristics and outcomes of patients with cattle-related trauma admitted to the trauma center of a tertiary hospital in China. The study results may contribute to the development of data-driven preventive measures for cattle-related trauma.

## 2. Methods

### 2.1. Ethics Approval

This study was approved by the Ethics Committee of 926th Hospital of Joint Logistics Support Force of People's Liberation Army (PLA) (No. 2022-128). Owing to the anonymization of the data and the retrospective nature of the current study, the need for informed consent was waived. The study was conducted in accordance with the principles of the Declaration of Helsinki.

### 2.2. Study Design

This retrospective cohort study was performed at a trauma center in China. During the 6-year period between October 1, 2016, and September 31, 2022, data of patients admitted to the hospital following cattle-related trauma were retrospectively analyzed.

### 2.3. Search Strategy and Eligibility Criteria

The Chinese refer to cattle, oxen, bulls, cows and buffalos, and other cattle-related terms as “牛” (niú), so we searched the Hospital Information System for this keyword and screened all medical records of patients admitted to our hospital within this particular period. Every medical record matching the keyword was manually checked to ensure that there was cattle-related trauma. Inclusion criterion is that all patients admitted to the hospital due to cattle-related trauma were included in this study. Exclusion criterion is that although the extracted medical records contained the Chinese word “牛,” they did not involve cattle-related trauma.

### 2.4. Data Collection

Demographic data were collected, including age, sex, and occupation of the trauma patients (e.g., farmer and teacher). To analyze the distribution of trauma patients in different age groups, patients were divided into four groups according to age: 0–17 years, 18–44 years, 45–64 years, and ≥ 65 years. The injury situation was extracted from the information recorded in the patient notes and stratified into the following groups: traveling in a bullock cart as a passenger, bullock cart driving as a driver, farm work, cattle leading, cattle pasturing, passing by, feeding, cattle fight, playing next to cattle, field ploughing, cattle riding, cattle catching, and unknown. The term passing by was used when no specific task or event was recorded at the time of injury [11, 14]. The mechanism of injury was confirmed from clinical notes and classified into several categories: charge/head-butt, horn goring, kick, trampling, bullock cart accident, rope injury, crushing, and others.

The injury severity was quantitatively evaluated using trauma scoring systems. The abbreviated injury scale (AIS) for each injury was coded in every patient. According to the AIS, body regions are divided into head (cranium and brain); face, including eyes and ears; neck; thorax; abdomen and pelvic contents; spine (cervical, thoracic, and lumbar); upper extremity; lower extremity, pelvis and buttocks; external (skin) and thermal injuries, and other trauma [15]. Severe injury was defined as AIS ≥ 3 [2, 16, 17]. The injury severity score (ISS) [18] of each patient was calculated using their AIS values. The ISS is the most commonly used scoring system for the overall evaluation of trauma patients by far [19]. Usually, major trauma is defined as an ISS ≥ 16 [20]. Data on surgical procedures were extracted from the clinical notes, discharge notes, and operation notes. Conservative treatment was considered to have been applied if no surgical procedure was performed. Patient outcomes, including length of hospital stay (LOS), intensive care unit (ICU) admission, length of ICU stay, and mortality, were collected.

### 2.5. Statistical Analysis

The qualitative variables were presented as frequencies and percentages. For continuous data, normality analysis was performed using the Kolmogorov–Smirnov test or Shapiro–Wilk test. If continuous data were not normally distributed, median and interquartile range (IQR) were computed. The Mann–Whitney *U* test was used for the statistical comparison of data that were not normally distributed. Statistical analyses were performed using the Statistical Package for the Social Sciences (SPSS) version 20. A two-tailed *p* < 0.05 was considered statistically significant.

## 3. Results

**Figure 1:**
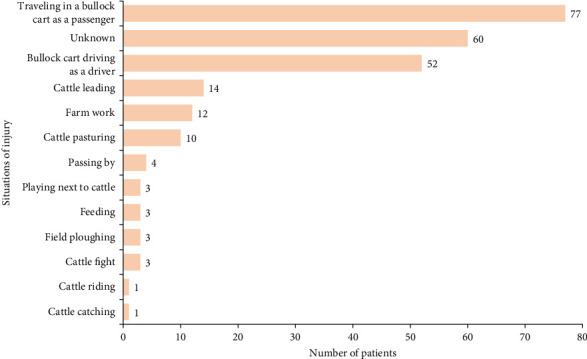
Situations of injury.

A total of 243 patients with cattle-related trauma were admitted to the hospital between October 1, 2016, and September 31, 2022. Overall, the median age was 48 years (IQR 31–57), ranging from 1 to 82 years. The age groups (0–17 years group, 18–44 years group, 45–64 years group, and ≥ 65 years group) comprised 28 (11.5%), 72 (29.6%), 116 (47.7%), and 27 (11.1%) patients, respectively. The study included 164 men (67.5%) and 79 women (32.5%) (Table 1). The most common occupation was farmer (88.1%, *n* = 214). Among the patients with specific injury situations recorded, traveling in a bullock cart as a passenger (31.7%, *n* = 77) and bullock cart driving as a driver (21.4%, *n* = 52) were the most frequent (Figure 1). The situation of injury in 24.7% (*n* = 60) of the patients was unknown. Bullock cart accident (57.6%, *n* = 140) was the most common mechanism of injury, followed by horn goring (18.1%, *n* = 44) and charge/head-butt (11.5%, *n* = 28) (Table 2).

Blunt trauma occurred in the majority of patients (86.4%, *n* = 210), and the injury mechanisms consisted of all eight types mentioned above, of which the three most common were bullock cart accident (63.3%, *n* = 133), charge/head-butt (11.4%, *n* = 24), and horn goring (11.0%, *n* = 23). Penetrating trauma accounted for 13.6% (*n* = 33) of cases, and the mechanisms involved were horn goring (63.6%, *n* = 21), bullock cart accident (21.2%, *n* = 7), charge/head-butt (12.1%, *n* = 4), and trampling (3.0%, *n* = 1) (Table 2). The median ISS for blunt trauma was 8 (IQR 4–13) compared to that for penetrating trauma 9 (IQR 4–13) (*p* = 0.900).

Generally, the most commonly injured body region was the lower extremity, pelvis and buttocks (38.3%, *n* = 93), followed by the upper extremity (35.0%, *n* = 85) and thorax (25.9%, *n* = 63) (Table 3). The most commonly injured body region in bullock cart accident, trampling, kick, and crushing was found to be the lower extremity, pelvis and buttocks. The thorax was the most commonly injured body region for both the horn goring and charge/head-butt mechanisms. Thirteen patients (5.3%) were injured by ropes, of whom 12 (92.3%) had hand injuries (including 8 patients with partial or complete traumatic amputation of the thumbs or fingers) and 1 (7.7%) had shoulder injury (Table 3).

Among the patients, 111 (45.7%) had at least one AIS of ≥ 3, and the mechanisms of injury were bullock cart accident (53.2%, *n* = 59), horn goring (25.2%, *n* = 28), charge/head-butt (17.1%, *n* = 19), trampling (2.7%, *n* = 3), kick (0.9%, *n* = 1), and crushing (0.9%, *n* = 1), respectively (Table 4). Patients with at least one severe injury accounted for 42.1% (59 of 140) of all patients injured by bullock cart accident, 63.6% (28 of 44) by horn goring, 67.9% (19 of 28) by charge/head-butt, 33.3% (3 of 9) by trampling, 25.0% (1 of 4) by kick, and 50.0% (1 of 2) by crushing. The thorax was the most common body region to experience at least one severe injury (AIS ≥ 3), followed by lower extremity, pelvis and buttocks, and head. 55 patients (49.5%) had at least one thorax AIS of ≥ 3, of which 22 patients (40.0%) were injured by horn goring, 17 (30.9%) by bullock cart accident, 14 (25.5%) by charge/head-butt, and 2 (3.6%) by trampling. Among the patients, 33 (29.7%) had at least one lower extremity, pelvis and buttocks AIS of ≥ 3, of which 27 patients (81.8%) were injured by bullock cart accident, 2 (6.1%) by horn goring, and the other 4 by kick (3.0%, *n* = 1), charge/head-butt (3.0%, *n* = 1), crushing (3.0%, *n* = 1), and trampling (3.0%, *n* = 1), respectively. Eleven patients (9.9%) had at least one head AIS of ≥ 3, of which 9 patients (81.8%) were injured by bullock cart accident, 1 (9.1%) by horn goring, and 1 (9.1%) by charge/head-butt (Table 4).

Fourteen (5.8%) patients had combined severe injuries in both body regions. Among these patients, three (21.4%) had both severe head (head AIS ≥ 3) and thoracic injuries (thorax AIS ≥ 3); combined severe thoracic (thorax AIS ≥ 3) and spinal injuries (spine AIS ≥ 3) were present in three (21.4%); combined severe thoracic (thorax AIS ≥ 3) and facial injuries (face AIS ≥ 3) were present in three (21.4%); combined severe thoracic (thorax AIS ≥ 3) and abdomen and pelvic contents injuries (abdomen and pelvic contents AIS ≥ 3) were present in two (14.3%); one (7.1%) had combined severe head and facial injuries (both AIS ≥ 3); one (7.1%) experienced combined severe lower extremity, pelvis and buttocks and facial injuries (both AIS ≥ 3); and one (7.1%) sustained combined severe injuries in lower extremity, pelvis and buttocks and thorax (both AIS ≥ 3).

The ISS of all 243 patients ranged from 1 to 45, with a median ISS was 8 (IQR 4–13). Horn goring was associated with the highest median ISS of 11.5 (IQR 4.25–17), while rope injury was associated with the lowest (ISS 2, IQR 1–3). Among the patients, 100 (41.2%) had an ISS ≤ 5, and 39 patients (16.0%) had an ISS ≥ 16 (major trauma). Of the 39 patients with ISS ≥ 16, 16 (41.0%) were injured by bullock cart accident, 13 (33.3%) by horn goring, 9 (23.1%) by charge/head-butt, and 1 (2.6%) by trampling. Patients with major trauma accounted for 11.4% (16 of 140) of all patients injured by bullock cart accident, 29.5% (13 of 44) by horn goring, 32.1% (9 of 28) by charge/head-butt, and 11.1% (1 of 9) by trampling.

The median age of patients with major trauma was 50 years (IQR 40–57) compared to 48 years (IQR 29–56.75) in those without major trauma (*p*=0.296). Of the patients with major trauma, men accounted for 56.4% (22 of 39) compared to 69.6% (142 of 204) of the patients without major trauma (*p*=0.107). The median LOS in patients with major trauma was 15 days (IQR 10–23) compared to 9 days (IQR 6–16) in patients without major trauma (*p*=0.001). Of the patients with major trauma, ICU admission was required in 100% (39 of 39 patients) compared to 14.7% (30 of 204 patients) without major trauma (*p* < 0.001).

**Figure 2:**
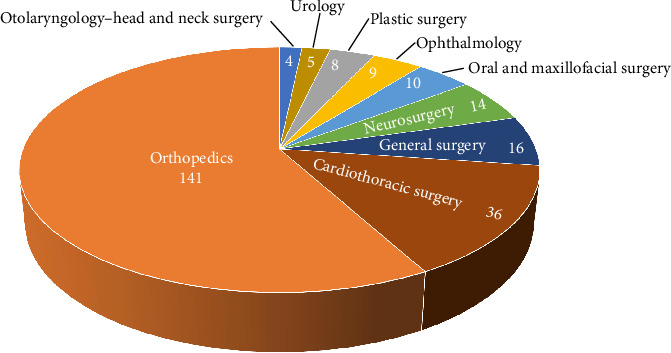
Admitting specialities.

**Figure 3:**
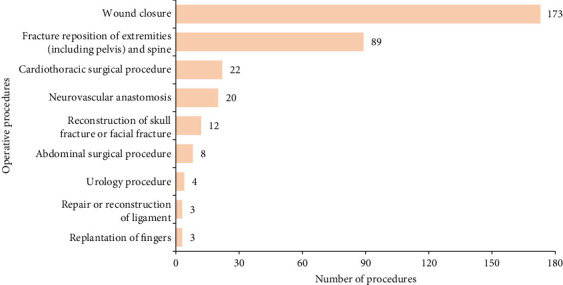
Operative procedures.

The two specialities where most patients were admitted to were orthopedics (58.0%, *n* = 141) and cardiothoracic surgery (14.8%, *n* = 36) (Figure 2). Conservative treatment was administered to 34 (14.0%) patients. A total of 334 operative procedures were performed on 209 patients (86.0%). Wound closure (51.8%, *n* = 173) was the most frequently performed procedure, followed by fracture reposition of the extremities (including the pelvis) and the spine (26.6%, *n* = 89) (Figure 3).

For the entire sample, the median LOS was 10 days (IQR 6–18). There were 131 patients (53.9%) with extended LOS (≥ 10 days) [21]. The median ISS of patients with extended LOS was significantly higher than that of patients without extended LOS (ISS 10, IQR 5–14 vs. ISS 5, IQR 3.25–9.75; *p* < 0.001). Overall, 69 patients (28.4%) were admitted to the ICU. The median length of ICU stay for the 69 patients admitted to the ICU was 4 days (IQR 3–9). Among the patients admitted to the ICU, 7 (10.1%) had a prolonged ICU stay (length of ICU stay > 14 days) [22]. Three male patients died (a mortality rate of 1.2%). The first patient (71 years old) was injured by horn goring and had severe thoracic injuries combined with external injuries (ISS 10). The second patient (64 years old) was injured by horn goring and had severe thoracic injuries combined with abdominal and spinal injuries (ISS 21). The third patient (55 years old), who had hepatocellular carcinoma prior to being injured, was injured in the abdomen by a charge/head-butt, leading to hemorrhage of the tumor (ISS 8).

## 4. Discussion

In this retrospective study, we showed the details of the injury characteristics and outcomes of patients with cattle-related trauma admitted to a trauma center in China. In our study, 243 patients were identified over 6 years. In line with previous literature, the prevalence of cattle-related trauma appears to have been underestimated [2, 14]. This is because not all patients with cattle-related trauma are admitted to trauma centers. Patients with cattle-related trauma admitted to a trauma center usually require advanced trauma care or surgical treatment (86.0% of the hospitalized patients received surgical treatment in the present study). Some patients with minor traumatic injuries are treated at local clinics or primary hospitals.

The demographics of the patients in the present study were consistent with those of two other studies; the majority of patients were middle-aged male farmers [2, 14]. In the present study, the median age was 48 years (IQR 31–57), and 116 patients (47.7%) were in the 45–64 years group.

Cattle-related injuries can have a wide range of types and severities and can be caused by several different mechanisms [2, 3]. This study supports the literature by finding that blunt trauma was more prevalent than penetrative trauma [2, 11]. However, our results showed that the severity of blunt and penetrating trauma was equivalent. In the present study, 41.2% (*n* = 100) of all the patients had an ISS ≤ 5, and 16.0% (*n* = 39) had an ISS ≥ 16; a study from Switzerland reported the corresponding indices as 63% and 12% [2].

All patients enrolled in this study lived in rural areas, and when injured, they were performing different types of activities. Among several known situations, traveling in a bullock cart as a passenger was the most common situation of injury, and the next most common was bullock cart driving as a driver. Bullock carts are a socially and culturally acceptable mode of transportation in rural areas of developing countries [23]. It is a relatively sluggish form of transportation; however, when animals are driven in them, unpredictable and aggressive behavior can occur, posing a serious risk to the driver and passengers [23]. As indicated in previous literature, trauma due to bullock cart accidents has been reported in India [23–25].

One review referred to kicking as the most prevalent mechanism of injury, followed by pushing and head-butting [3]. In our study, the three most common mechanisms were bullock cart accident, horn goring, and charge/head-butt. Among all patients with charge/head-butt injuries, 67.9% (19 of 28 patients) had at least one severe injury, and this proportion was the highest among all injury mechanisms; 32.1% (9 of 28 patients) had major trauma, and this proportion was also the highest among all injury mechanisms. From the perspective of causing severe injury and major trauma, the charge/head-butt is the most dangerous mechanism.

Bullock cart accident was the most common mechanism of injury in all patients, in patients with at least one severe injury (AIS ≥ 3) and in patients with major trauma (ISS ≥ 16) in our study. As mentioned above, the bullock cart accident was reported as an injury mechanism in the studies from India [23–25]. In other previous studies on cattle-related trauma, the bullock cart was not reported as a situation or mechanism of injury [1, 2, 11, 14]. The reason for this discrepancy is that the purposes of cattle raising and patterns of the population included in studies from different countries vary; therefore, the mechanisms and situations of injury vary. As mentioned in studies from New Zealand, Switzerland, and England, cattle in these countries are mainly raised as dairy cows and beef cattle [1, 2, 11]. Besides large cattle and dairy farms raising cattle, some individual farmers also raise cattle to help with farm work, such as pulling carts and ploughing fields. Thus, a bullock cart accident was reported as a mechanism of injury in previous studies [23–25].

Horn goring was the second most common mechanism of injury in all patients, patients with at least one severe injury, and patients with major trauma. Among the patients injured by horn goring, 47.7% (21 of 44) sustained penetrating trauma and horn goring accounted for 63.6% (21 of 33 patients) of penetrating trauma. Of the patients injured by horn goring, 29.5% (13 of 44) had major trauma, and horn goring accounted for 33.3% (13 of 39 patients) of major trauma. These results show that a significant number of penetrating and major traumas were caused by horn goring. Among all the mechanisms in this study, horn goring had the highest ISS (median 11.5). Furthermore, in our study, horn goring was the most common mechanism of severe thoracic injuries (22 of the 55 patients with at least one thoracic AIS of ≥ 3). Two of the three dead patients in this study were injured by horn goring. In another study that included 63 cattle-related trauma patients, the only death caused by cattle occurred after the individual was gored by a bull [26]. These findings indicate that cattle horns are dangerous to farmers. Therefore, dehorning is important to avoid cattle-related trauma. Dehorning is performed for safety purposes in 61% of cattle herds in Europe and 73% of dairy cows in Switzerland [2]. Ehrhard et al. [2] suggested that the widespread use of cattle dehorning contributes to a reduction in penetrating injuries.

Although associated with the lowest ISS (median 2, IQR 1–3), rope injury is another mechanism that requires attention. Our results showed that 8 of the 13 patients injured by a rope had partial or complete traumatic amputation of the thumbs or fingers. A previous study reported that thumb amputations resulting from the leading cattle had a lower chance of successful replantation than those resulting from other causes [27]. Though the AIS of traumatic amputation of hand, thumb, and fingers (score of 1 or 2) does not belong to severe injury (AIS ≥ 3), this kind of injury impairs hand function, creates disability, and has a negative influence on the patients' quality of life and capacity to work.

Our findings indicated that the most commonly injured body region was lower extremity, pelvis and buttocks, followed by upper extremity and thorax. These findings are similar to those of previous studies, which indicated that the most common injuries were those affecting the upper and lower extremities [3]. The study conducted by Murphy et al. in Ireland including 63 patients after cattle-related trauma also reported that injuries to the lower and upper extremities were the most common, followed by blunt chest injuries [3, 14]. Another study conducted by Norwood et al. [26] reported results different from the findings described above: the torso (chest/abdomen/pelvis), followed by the brain and craniofacial areas. We consider the reason for these differences to be the different situations in which people are injured and the different injury mechanisms. In the research conducted by Norwood et al. [26], the most common mechanisms were being stepped on/trampled and fall/thrown, whereas in the research performed by Murphy et al. [14], the most common mechanisms were the kick and charge/head-butt, and in our study, the most common mechanisms were bullock cart accident, horn goring, and charge/head-butt.

Our findings also indicate that different body regions are injured by different mechanisms. The two most common body regions injured in bullock cart accidents are lower extremity, pelvis and buttocks, and upper extremity. This may be because of the protective movements of the limbs in such accidents to protect the torso, head, and neck from getting injuries. Horn goring mainly injures the thorax and the abdomen and pelvic contents, and the most common body region injured by the charge/head-butt was the thorax. This may be because the cattle horns, human thorax, and abdomen have similar heights, and the height of the cattle head is approximately equivalent to that of the human thorax in the standing position when a charge/head-butt occurs. Therefore, these two mechanisms are prone to injure the corresponding body regions. Rope injuries were all in the upper extremity because ropes are pulled using the upper extremity. Both trampling and kick are carried out by the legs of cattle, mainly injuring the lower extremity, pelvis and buttocks, as the range of cattle trampling and kicking is mainly equivalent to that of the lower extremity, pelvis and buttocks of humans.

Although the number of patients with thoracic injury ranked third among the number of patients categorized by injured body region, the number of patients with at least one severe injury in the thorax was higher than that of patients with at least one severe injury in any other body region. The reason for this may be the concentration of vital organs in the thorax, which is prone to severe injuries after a vital organ is injured. Therefore, when dealing with patients injured by horn goring and charge/head-butt, especially in pre-hospital emergency care, special attention should be paid to the presence of severe thoracic injuries. Among the body regions with at least one severe injury caused by a bullock cart accident, the top three were the lower extremity, pelvis and buttocks, thorax, and head. Therefore, when treating patients injured by this mechanism, a thorough examination should be performed for the presence of severe injuries to the lower extremity, pelvis and buttocks, thorax, and head.

The upper and lower extremity injuries are associated with high patient morbidity, whereas torso and head injuries are related to high patient mortality [2, 3]. Older male farmers with blunt head and chest injuries have been identified as a high-risk group for cattle-related deaths [2, 11, 28]. Our data were consistent with these findings. In this study, the thorax and head were included in the three most common body regions that sustained severe injuries (the other is lower extremity, pelvis and buttocks). The more severe the injury, the higher is the risk of death. The 3 patients who died were all male farmers with injuries in torso. Two of the three patients without preexisting morbidities were aged 71 years and 64 years, respectively, and both sustained severe blunt thoracic injuries. One patient was 55 years old with preexisting morbidity of hepatocellular carcinoma and sustained blunt abdominal injuries, leading to hemorrhage of the hepatocellular carcinoma. The results reported by Rhind et al. [11] also showed that the two deaths in their study occurred in male farmers over 50 years of age. Karbeyaz et al. [4] reported that 71.4% of deaths were caused by chest injuries.

In this study, orthopedics was the most common speciality the patients were admitted under, followed by cardiothoracic surgery. This result is consistent with that of a previous study conducted in England [11]. In the present study, 209 patients underwent 334 operative procedures, of which wound closure was the most frequently performed, followed by fracture reposition. This finding was consistent with that of a previous study [2]. In our study, the LOS of patients was 10 days (IQR 6–18); Ehrhard et al. [2] reported an LOS of 4.5 days (IQR 3–8); Norwood et al. [26] reported an LOS of 7.0 ± 8.1 days for bull-related trauma and an LOS of 6.4 ± 3.7 days for cow-related trauma; San Norberto et al. [29] reported an LOS of 11.7 ± 9.4 days for bull horn vascular injuries. Length of ICU stay of patients admitted to the ICU in our study was 4 days (IQR 3–9). Sheehan et al. [30] reported an length of ICU stay of 4 days, and San Norberto et al. [29] reported an length of ICU stay of 4.5 ± 11.3 days.

Our study may help propose recommendations for preventive measures to handle cattle safely, thus reducing the morbidity and mortality of cattle-related trauma. It is important to understand animal behavior and obtain training on safety when driving a bullock cart. Cattle horns are dangerous to humans, and cattle should be dehorned for safety. When working with, around, or in contact with cattle, it is essential to wear protective gear, including helmets, protective clothing, and protective gloves. Safety education, guidelines, and regulations for raising, handling, and working with cattle need to be implemented to minimize the risk of cattle injury. Promoting a registration system for cattle-related trauma in local rural clinics and hospitals at all levels may help monitor and comprehensively understand this type of trauma. Introducing specific coding for cattle-related trauma into the International Classification of Diseases (ICD) would make research in this field more convenient and effective [3, 11].

## 5. Strengths and Limitations

To our knowledge, this is the first study to evaluate cattle-related trauma in China, bridging the gap in the literature. Another strength is that this study included all consecutive admissions for cattle-related trauma at a large trauma center in China. The sample size of this study was larger than that of other studies in the same category, which could provide more details on trauma characteristics. However, the study design was retrospective, relating to the native limitations of this type of design. Another limitation is that this study was performed at a single center and included only in-hospital patients, which may not address the characteristics of cattle-related trauma in outpatient cases.

## 6. Conclusion

The present study demonstrated the characteristics of cattle-related trauma in a trauma center in China. Cattle-related trauma is under-reported, and it requires more attention. The results of this study can help propose data-driven safety precautions to reduce the risk of cattle-related trauma.

## Figures and Tables

**Table 1:** Patient characteristics by demographic subgroups.

Demographics	Total^a^ (*n* = 243)	Patient characteristics				
		ISS^b^	Patients with ISS ≥ 16^a^ (*n* = 39)	LOS (days)^b^	ICU admission^a^ (*n* = 69)	Death^a^ (*n* = 3)
Sex						
Male	164 (67.5)	8.5 (4–13)	22 (56.4)	10.5 (6–17.75)	48 (69.6)	3 (100.0)
Female	79 (32.5)	8 (5–14)	17 (43.6)	10 (5–18)	21 (30.4)	0 (0.0)
Age group (years)						
0–17	28 (11.5)	5 (5–10)	4 (10.3)	7.5 (4.25–16)	5 (7.2)	0 (0.0)
18–44	72 (29.6)	8 (4–13)	7 (17.9)	10 (6–16)	12 (17.4)	0 (0.0)
45–64	116 (47.7)	9 (4–13.75)	25 (64.1)	11 (6–19)	42 (60.9)	2 (66.7)
≥ 65	27 (11.1)	9 (2–13)	3 (7.7)	12 (6–16)	10 (14.5)	1 (33.3)

Abbreviations: ICU, intensive care unit; ISS, injury severity score; LOS, length of hospital stay.

^a^Data were presented as *n* (%).

^b^Data were presented as median (interquartile range).

**Table 2:** Characteristics of injury mechanisms.

Injury mechanisms	Total^a^ (*n* = 243)	Characteristics			
		Blunt trauma^a^ (*n* = 210)	Penetrating trauma^a^ (*n* = 33)	ISS^b^	Patients with ISS ≥ 16^a^ (*n* = 39)
Bullock cart accident	140 (57.6)	133 (63.3)	7 (21.2)	8 (5–12.75)	16 (41.0)
Horn goring	44 (18.1)	23 (11.0)	21 (63.6)	11.5 (4.25–17)	13 (33.3)
Charge/head-butt	28 (11.5)	24 (11.4)	4 (12.1)	9.5 (5.25–17.75)	9 (23.1)
Rope injury	13 (5.3)	13 (6.2)	0 (0.0)	2 (1–3)	0 (0.0)
Trampling	9 (3.7)	8 (3.8)	1 (3.0)	5 (1.5–10)	1 (2.6)
Kick	4 (1.6)	4 (1.9)	0 (0.0)	4.5 (2.5–8.75)	0 (0.0)
Crushing	2 (0.8)	2 (1.0)	0 (0.0)	10.5 (– –)^c^	0 (0.0)
Others	3 (1.2)	3 (1.4)	0 (0.0)	4 (– –)^c^	0 (0.0)

Abbreviation: ISS, injury severity score.

^a^Data were presented as *n* (%).

^b^Data were presented as median (interquartile range).

^c^There were only two or three numbers in the dataset so that the interquartile range could not be calculated by the software SPSS.

**Table 3:** Number of patients with specific body region injured from different mechanisms.

Body regions	Total (*n* = 243)	Mechanisms							
		Bullock cart accident (*n* = 140)	Horn goring (*n* = 44)	Charge/head-butt (*n* = 28)	Rope injury (*n* = 13)	Trampling (*n* = 9)	Kick (*n* = 4)	Crushing (*n* = 2)	Others (*n* = 3)
Head	34 (14.0)	23 (16.4)	6 (13.6)	5 (17.9)	0 (0.0)	0 (0.0)	0 (0.0)	0 (0.0)	0 (0.0)
Face	47 (19.3)	22 (15.7)	14 (31.8)	8 (28.6)	0 (0.0)	0 (0.0)	1 (25.0)	0 (0.0)	2 (66.7)
Neck	5 (2.1)	3 (2.1)	2 (4.5)	0 (0.0)	0 (0.0)	0 (0.0)	0 (0.0)	0 (0.0)	0 (0.0)
Thorax	63 (25.9)	22 (15.7)	23 (52.3)	16 (57.1)	0 (0.0)	2 (22.2)	0 (0.0)	0 (0.0)	0 (0.0)
Abdomen and pelvic contents	35 (14.4)	7 (5.0)	21 (47.7)	6 (21.4)	0 (0.0)	1 (11.1)	0 (0.0)	0 (0.0)	0 (0.0)
Spine	23 (9.5)	11 (7.9)	7 (15.9)	5 (17.9)	0 (0.0)	0 (0.0)	0 (0.0)	0 (0.0)	0 (0.0)
Upper extremity	85 (35.0)	54 (38.6)	7 (15.9)	8 (28.6)	13 (100.0)	1 (11.1)	1 (25.0)	0 (0.0)	1 (33.3)
Lower extremity, pelvis and buttocks	93 (38.3)	68 (48.6)	10 (22.7)	4 (14.3)	0 (0.0)	7 (77.8)	2 (50.0)	2 (100.0)	0 (0.0)
External	33 (13.6)	17 (12.1)	7 (15.9)	5 (17.9)	0 (0.0)	3 (33.3)	0 (0.0)	1 (50.0)	0 (0.0)

*Note:* Data were presented as *n* (%).

**Table 4:** Number of patients with at least one AIS ≥ 3 in specific body region injured.

Mechanisms	Total (*n* = 111)	Number of patients with at least one AIS ≥ 3									
		Thorax (*n* = 55)	Lower extremity, pelvis and buttocks (*n* = 33)	Head (*n* = 11)	Face (*n* = 9)	Spine (*n* = 7)	Abdomen and pelvic contents (*n* = 5)	Upper extremity (*n* = 5)	Neck (*n* = 0)	External (*n* = 0)	Patients with combined severe injuries (AIS ≥ 3) in both body regions (*n* = 14)
Bullock cart accident	59 (53.2)	17 (30.9)	27 (81.8)	9 (81.8)	3 (33.3)	4 (57.1)	2 (40.0)	4 (80.0)	0 (0.0)	0 (0.0)	7 (50.0)
Horn goring	28 (25.2)	22 (40.0)	2 (6.1)	1 (9.1)	3 (33.3)	1 (14.3)	1 (20.0)	0 (0.0)	0 (0.0)	0 (0.0)	2 (14.3)
Charge/head-butt	19 (17.1)	14 (25.5)	1 (3.0)	1 (9.1)	3 (33.3)	2 (28.6)	2 (40.0)	1 (20.0)	0 (0.0)	0 (0.0)	5 (35.7)
Rope injury	0 (0.0)	0 (0.0)	0 (0.0)	0 (0.0)	0 (0.0)	0 (0.0)	0 (0.0)	0 (0.0)	0 (0.0)	0 (0.0)	0 (0.0)
Trampling	3 (2.7)	2 (3.6)	1 (3.0)	0 (0.0)	0 (0.0)	0 (0.0)	0 (0.0)	0 (0.0)	0 (0.0)	0 (0.0)	0 (0.0)
Kick	1 (0.9)	0 (0.0)	1 (3.0)	0 (0.0)	0 (0.0)	0 (0.0)	0 (0.0)	0 (0.0)	0 (0.0)	0 (0.0)	0 (0.0)
Crushing	1 (0.9)	0 (0.0)	1 (3.0)	0 (0.0)	0 (0.0)	0 (0.0)	0 (0.0)	0 (0.0)	0 (0.0)	0 (0.0)	0 (0.0)
Others	0 (0.0)	0 (0.0)	0 (0.0)	0 (0.0)	0 (0.0)	0 (0.0)	0 (0.0)	0 (0.0)	0 (0.0)	0 (0.0)	0 (0.0)

*Note:* Data were presented as *n* (%).

Abbreviation: AIS, abbreviated injury scale.

## Data Availability

The data that support the findings of this study are available from the corresponding author upon reasonable request.
